# Early factors associated with continuous positive airway pressure failure in moderate and late preterm infants

**DOI:** 10.1007/s00431-023-05090-1

**Published:** 2023-09-26

**Authors:** Pierre Tourneux, Thierry Debillon, Cyril Flamant, Pierre-Henri Jarreau, Benjamin Serraz, Isabelle Guellec

**Affiliations:** 1 Neonatal Intensive Care Unit, University Hospital Center of Amiens, Jules Verne University of Picardy, Amiens, France; 2 Neonatology Intensive Care Unit, University Hospital of Grenoble, Grenoble, France; 3 Neonatal Intensive Care Unit, University Hospital of Nantes, Nantes, France; 4 Neonatal Intensive Care Unit of Port-Royal, AP–HP,, University of Paris , Paris, France; 5 Medical Affairs, Chiesi SAS, Bois Colombes, France; 6 Neonatal and Paediatric Intensive Care Unit, University Hospital of L’Archet, Nice, France

**Keywords:** CPAP failure, Fraction of inspired oxygen, Late preterm infant, Positive end-expiratory pressure, Predictive value, Respiratory distress syndrome

## Abstract

To determine the early factors associated with continuous positive airway pressure (CPAP) failure in moderate-to-late preterm infants (32 + 0/7 to 36 + 6/7 weeks’ gestation) from the NEOBS cohort study. The NEOBS study was a multi-center, prospective, observational study in 46 neonatal intensive care units in France, which included preterm and late preterm infants with early neonatal respiratory distress. This analysis included a subset of the NEOBS population who had respiratory distress and required ventilatory support with CPAP within the first 24 h of life. CPAP failure was defined as the need for tracheal intubation within 72 h of CPAP initiation. Maternal and neonatal clinical parameters in the delivery room and clinical data at 3 h of life were analyzed. CPAP failure occurred in 45/375 infants (12%), and compared with infants with CPAP success, they were mostly singletons (82.2% vs. 62.1%; *p* < 0.01), had a lower Apgar score at 10 min of life (9.1 ± 1.3 vs. 9.6 ± 0.8; *p* = 0.02), and required a higher fraction of inspired oxygen (FiO_2_; 34.4 ± 15.9% vs. 22.8 ± 4.1%; *p* < 0.0001) and a higher FiO_2_*positive end-expiratory pressure (PEEP) (1.8 ± 0.9 vs. 1.1 ± 0.3; *p* < 0.0001) at 3 h. FiO_2_ value of 0.23 (*R*^2^ = 0.73) and FiO_2_*PEEP of 1.50 (*R*^2^ = 0.75) best predicted CPAP failure. The risk of respiratory distress and early CPAP failure decreased 0.7 times per 1-week increase in gestational age and increased 1.7 times with every one-point decrease in Apgar score at 10 min and 19 times with FiO_2_*PEEP > 1.50 (vs. ≤ 1.50) at 3 h (*R*^2^ of the overall model = 0.83).

*  Conclusion*: In moderate-to-late preterm infants, the combination of singleton pregnancy, lower Apgar score at 10 min, and FiO_2_*PEEP > 1.50 at 3 h can predict early CPAP failure with increased accuracy.

**Table:** 

**What is Known:**
•*Respiratory distress syndrome (RSD) represents an unmet medical need in moderate-to-late preterm births and is commonly treated with continuous positive airway pressure (CPAP) to reduce mortality and the need for additional ventilatory support.*
• *Optimal management of RSD is yet to be established, with several studies suggesting that identification of predictive factors for CPAP failure can aid in the prompt treatment of infants likely to experience this failure.*
**What is New:**
•*Secondary analysis of the observational NEOBS study indicated that oxygen requirements during CPAP therapy, especially the product of fraction of inspired oxygen (FiO2) and positive end-expiratory pressure (PEEP), are important factors associated with early CPAP failure in moderate-to-late term preterm infants.*
•The combination of a singleton pregnancy, low Apgar score at 10 minutes, and high FiO2*PEEP at 3 hours can predict early CPAP failure with increased accuracy, highlighting important areas for future research into the prevention of CPAP failure.

**Supplementary Information:**

The online version contains supplementary material available at 10.1007/s00431-023-05090-1.

## Introduction

Moderate and late preterm births, occurring at 32–33 or 34–36 weeks of gestation (WG), respectively, account for the vast majority (≈85%) of all preterm births, representing about 13 million preterm infants per year worldwide [1].

While less common in moderate and late preterm infants compared with infants born extremely or very preterm, respiratory distress syndrome (RDS) is still an important issue in this population [2, 3]. In a contemporary cohort of late preterm and term births, late preterm birth was associated with increased risk for RDS and other respiratory morbidities [4].

Treatment with continuous positive airway pressure (CPAP) has been shown to reduce mortality and the need for additional ventilatory support in preterm infants with respiratory distress [5]. Immediate initiation of nasal CPAP in the delivery room is recommended for all spontaneously breathing preterm infants with any clinical sign suggesting RDS [6–8]. Initial positive end-expiratory pressure (PEEP) should be started at 6–8 cmH_2_O and then individually titrated based on clinical condition, oxygenation, and perfusion [6, 7]. However, the level and quality of evidence for these recommendations are moderate to low, and optimal management of RDS in moderate-to-late preterm infants is yet to be established. Consistent with the recommendations for RDS, widespread use of CPAP in moderate-to-late preterm infants in France was recently documented in the multi-center, prospective, observational NEOBS study [9]. In this cohort study, the most common etiologies of respiratory failure in the overall population were transient tachypnea (57.3%) and RDS (39.8%). Surfactant therapy was administered to 22.5% of the total population, and 16.4% required mechanical ventilation [9].

Several studies have specified that identification of predictive factors for CPAP failure would be useful for the prompt treatment of infants likely to experience CPAP failure [10–12]. Therefore, the aim of the present analysis was to identify early factors associated with CPAP failure in moderate-to-late preterm infants from the NEOBS cohort study.

## Methods

### Study design and population

The study design and participant eligibility criteria for the NEOBS study have been reported previously [9]. The NEOBS study was a multi-center, prospective, observational study that was conducted in 46 neonatal intensive care units (level 2 or 3) in France [9]. This study was performed in line with the principles of the Declaration of Helsinki. The NEOBS study received ethical approval from the West V Rennes Research Ethics Committee (Comité de Protection des Personnes, CPP) in October 2017 [9].

Study data were collected between 6 February 2018 and 28 November 2018. Infants were eligible for inclusion if they were born between 32 + 0/7 and 36 + 6/7 WG, had respiratory distress that required ventilatory support with CPAP within the first 24 h of life, were hospitalized at the investigating site within the first 24 h of life, and if informed consent to participate were obtained from either the parents or legal guardians. Infants were excluded if they required ventilatory support for an indication other than respiratory distress or a malformation disorder, required tracheal intubation prior to initiating CPAP treatment in the delivery room, died within the first 24 h, or were enrolled in a clinical trial involving ventilatory care impact. Infants with respiratory distress were treated according to current guidelines [6, 7]. Scheduled study visits occurred at 72 h (visit 1), day 7 (visit 2), and at hospital discharge to home (visit 3) or on day 60 (if the infant was still in hospital).

### Study objective

The objective was to identify early factors (within the first 3 h of life) associated with CPAP failure, defined as the need for tracheal intubation within 72 h of CPAP initiation. The variables analyzed were maternal and neonatal clinical parameters in the delivery room, as well as clinical data at 3 h of life.

### Assessments and data collection

Data were recorded by local investigators using an electronic case report form. Maternal, pregnancy, and delivery characteristics were recorded, as well as management in the delivery room, ventilatory support used, duration of ventilation, maximum fraction of inspired oxygen (FiO_2_) value, maximum PEEP value, the product of maximum FiO_2_ and PEEP (FiO_2_*PEEP), surfactant administration, and time to withdrawal of all respiratory support. Infants were followed up for at least 7 days after birth, until hospital discharge or until day 60 (if the infant was still in hospital).

### Statistical analysis

Descriptive analyses of qualitative variables comprise the number of patients and percentage for each category. Descriptive analyses of quantitative variables comprise mean and standard deviation. Logistic regression models were created to identify factors predictive of early CPAP failure based on variables of clinical interest. All potentially explanatory variables were tested in univariate analyses using the Student’s *t*-test if the normal distribution (Shapiro–Wilk test) and the assumption of homogeneity of variance were verified, the Satterthwaite method if the variances were unequal, or the Wilcoxon-Mann–Whitney non-parametric test if the assumptions were not verified. The comparison of a categorical variable between two independent groups was assessed with the Pearson’s Chi-square test. Variables with *p* < 0.20 were included in the multiple logistic regression model; stepwise selection was performed on adjusted variables to eliminate those with an overall *p* value > 0.05. Gestational weeks, instead of birth weight, were used for logistic regression as European neonatologists have a preference for gestational weeks. The performance of the logistic regression analysis model was evaluated by assessing the area under receiver operating characteristic (ROC) curves. The Hosmer–Lemeshow goodness-of-fit test was used, when required. Analyses were performed using the available data, with no imputation of missing data. SAS^®^ software (version 9.4, SAS Institute, North Carolina USA) was used for performing statistical analyses.

## Results

### Participants

**Fig. 1:**
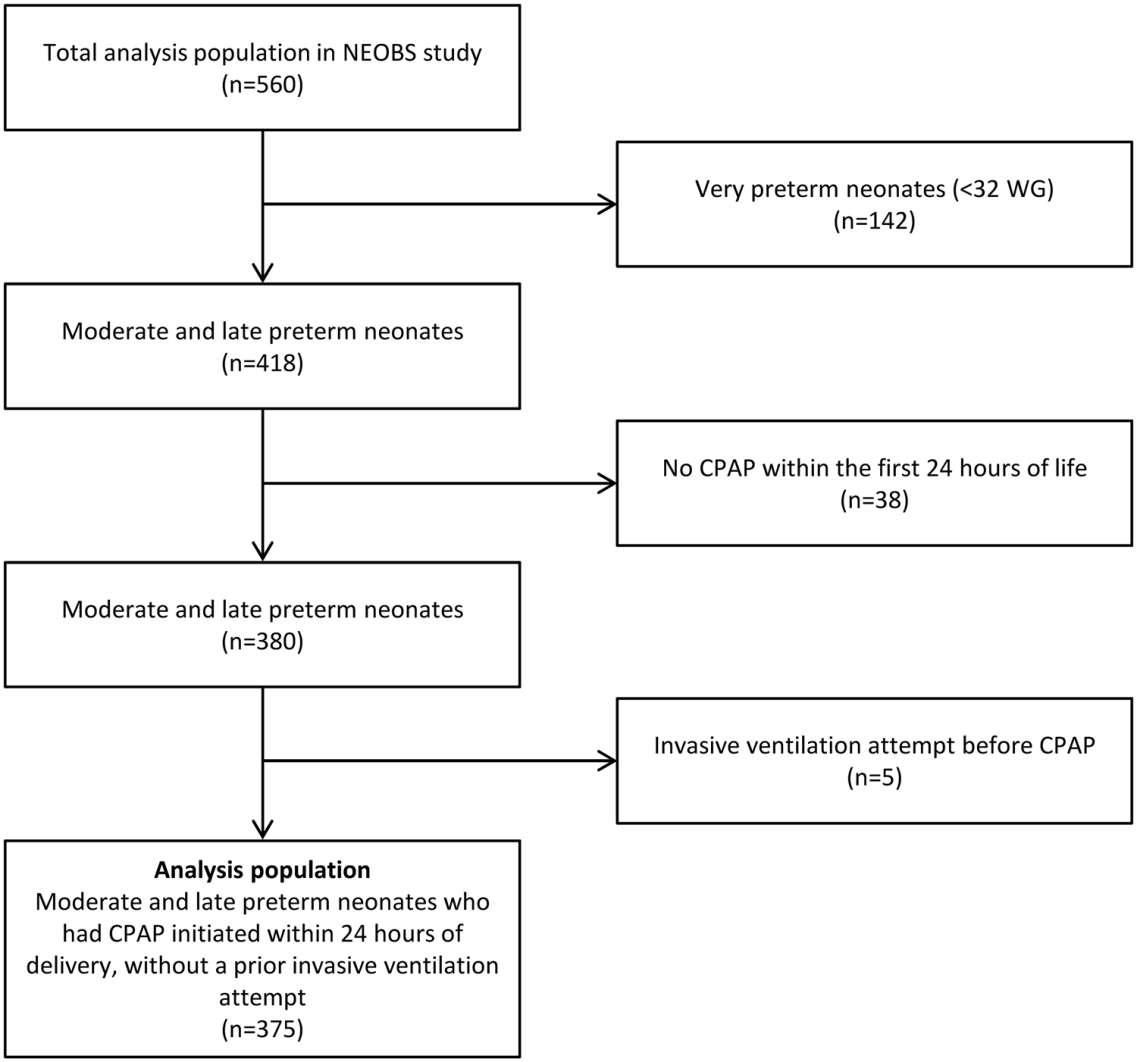
Patient inclusion flowchart. CPAP, continuous positive airway pressure; WG, weeks of gestation

Of the 560 participants in the main NEOBS study, 418 were moderate-to-late preterm infants and 375 were treated with CPAP within 24 h of delivery without a prior invasive ventilation attempt (Fig. 1). Of these, 121 infants (32.3%) were moderate preterm and 254 (67.7%) were late preterm.

### CPAP failure

CPAP failure occurred in 45/375 neonates (12%). There were notable differences in characteristics between infants with CPAP failure and CPAP success (Table 1). The proportion of singletons with CPAP failure was substantially higher than the proportion of twins who had CPAP failure. In the CPAP failure group, the proportion of newborn infants with an Apgar score < 7 at 5 min was higher (22.2% compared with 6.1% in the success group; *p* = 0.01) and the mean Apgar score at 10 min was lower (Table 1). Other significant differences between those with CPAP failure versus CPAP success included a higher maximum FiO_2_ at 3 h of life (Table 1 and Supplementary Table S1; Online Resource 1), a higher use of PEEP of ≥ 6 cmH_2_0 at 3 h of life, a more frequent pH of < 7.2 from 0 to 24 h, and a more frequent carbon dioxide pressure (pCO_2_) > 60 mmHg from 0 to 24 h. No differences were identified between clinically relevant characteristics, such as the Silverman score or intrauterine growth retardation. There were several significant differences in the time course of respiratory support between neonates with CPAP failure versus CPAP success (Supplementary Table S2; Online Resource 1). For example, the proportion of infants with maximum FiO2 > 21% was significantly higher among infants with CPAP failure than in those with CPAP success at all evaluated timepoints, 3, 6, 12, and 24 h of life.

**Table 1:** Characteristics of infants with early continuous positive airway pressure therapy success and failure (univariate analysis)

	**CPAP success (*n* = 330)**	**CPAP failure (*n* = 45**)	**Total analyzed population (*n*** **= 375)**	***p* value**
Delivery, *n* (%)				
Vaginal	131 (39.7)	18 (40.0)	149 (39.7)	0.97
Cesarean section	199 (60.3)	27 (60.0)	226 (60.3)	
Antenatal corticosteroids used, *n* (%)	196 (59.8)	25 (58.1)	221 (59.6)	0.84
Pregnancy type, *n* (%)				
Singleton	205 (62.1)	37 (82.2)	242 (64.5)	< 0.01
Multiple	125 (37.9)	8 (17.8)	133 (35.5)	
Delayed umbilical cord clamping, *n* (%)	43 (14.0)	5 (12.2)	48 (13.8)	0.61
Male sex, *n* (%)	195 (59.1)	27 (60.0)	222 (59.2)	0.91
Gestational age (weeks), mean ± SD Median [25^th^–75^th^ *P*]	34.0 ± 1.3 34 [33–35]	33.8 ± 1.3 34 [32–34]	33.9 ± 1.3 34 [33–35]	0.29
Birth weight (g), mean ± SD	2114.9 ± 510.6	2250.4 ± 513	2131.1 ± 512.1	0.10
Median [25^th^–75^th^ *P*]	2122.5 [1760–2445]	2180 [1902– 2615]	2135 [1780– 2460]	
IUGR, *n* (%)	28 (8.5)	1 (2.2)	29 (7.7)	0.23
Apgar score				
At 5 min mean ± SD Median [25^th^–75^th^ *P*]	9.1 ± 1.310 [8–10]	8.3 ± 2.29 [7–10]	9.0 ± 1.410 [8–10]	0.10
At 10 min mean ± SD Median [25^th^–75^th^ *P*]	9.6 ± 0.810 [9–10]	9.1 ± 1.310 [9–10]	9.5 ± 0.910 [9–10]	0.02
Respiratory parameters				
Max FiO_2_ at 3 h (%), mean ± SD	22.8 ± 4.1 21	34.4 ± 15.9	24.1 ± 7.6	< 0.0001
Highest Silverman score at 3 h, mean ± SD	3.5 ± 1.8	4.0 ± 2.1	3.6 ± 1.8	0.36
PEEPmax in delivery room, mean ± SD (cmH_2_O)	5.1 ± 1.2	5.0 ± 0.5	5.1 ± 1.1	0.97
PEEPmax at 3 h, mean ± SD (cmH_2_O)	4.9 ± 0.8	5.2 ± 0.9	4.9 ± 0.8	0.08
Min pH from 0 to 24 h, mean ± SD	7.3 ± 0.1	7.2 ± 0.1	7.3 ± 0.1	0.06
Max pCO_2_ from 0 to 24 h, mean ± SD (mmHg)	52.4 ± 10.3	54.6 ± 13.5	52.7 ± 10.8	0.35

*FiO*_*2*_ inspired oxygen fraction, *IUGR* intrauterine growth retardation (defined as weight ≤ 10% of the predicted fetal weight for gestational age), *max* maximum, *min* minimum, *pCO*_*2*_,partial pressure of carbon dioxide, *PEEPmax* maximum positive end-expiratory pressure with non-invasive ventilation

There were few notable differences in treatment approaches between infants with CPAP failure and CPAP success (Supplementary Table S3; Online Resource 1). A significantly higher proportion of infants with CPAP failure were administered surfactant within 24 h of birth (77.8% vs 6.4% with CPAP success; *p* < 0.0001).

### Cut-off for FiO_2_ and FiO_2_*PEEP associated with early CPAP failure

Optimal cut-off values for FiO_2_ and FiO_2_*PEEP at 3 h after delivery were determined from ROC curve analysis. The best *R*^2^ value (0.73) was found at a FiO_2_ cut-off value of 0.23, and an *R*^2^ value of 0.75 was found at a FiO_2_*PEEP cut-off value of 1.50 (Supplementary Fig. 1).

### Prediction of early CPAP failure

Table 2 presents results of the multivariate logistic model. Four early variables (those occurring in the first 3 h of life) were observed and entered in the backward logistic regression analysis.

**Table 2:** Logistic regression model to determine factors contributing to early failure of continuous positive airway pressure therapy

	**CPAP failure (*n*** **= 32) vs. CPAP success (*n* = 202)**		
	**Odds ratio (95% CI)**	***p* value**	**Area under ROC curve**
Gestational age^a^ (per 1-week increase)	0.703 (0.493–1.004)	0.0526	0.8304
Apgar score at 10 min (per 1-point decrease)	1.725 (1.148–2.591)	0.0086	
FiO_2_*PEEP at 3 h (reference ≤ 1.50)	18.660 (7.158–48.640)	< 0.0001	

*CI* confidence interval, *CPAP* continuous positive airway pressure, *FiO*_*2*_ fraction of inspired oxygen, *PEEP* positive end-expiratory pressure, *ROC* receiver operating characteristics

^a^Adjustment for gestational age

Of the four variables entered in the logistic regression analysis (gestational age, type of pregnancy, Apgar score at 10 min, and FiO_2_*PEEP at 3 h), gestational age was forced and only type of pregnancy was not retained in the final model (Table 2).

**Fig. 2:**
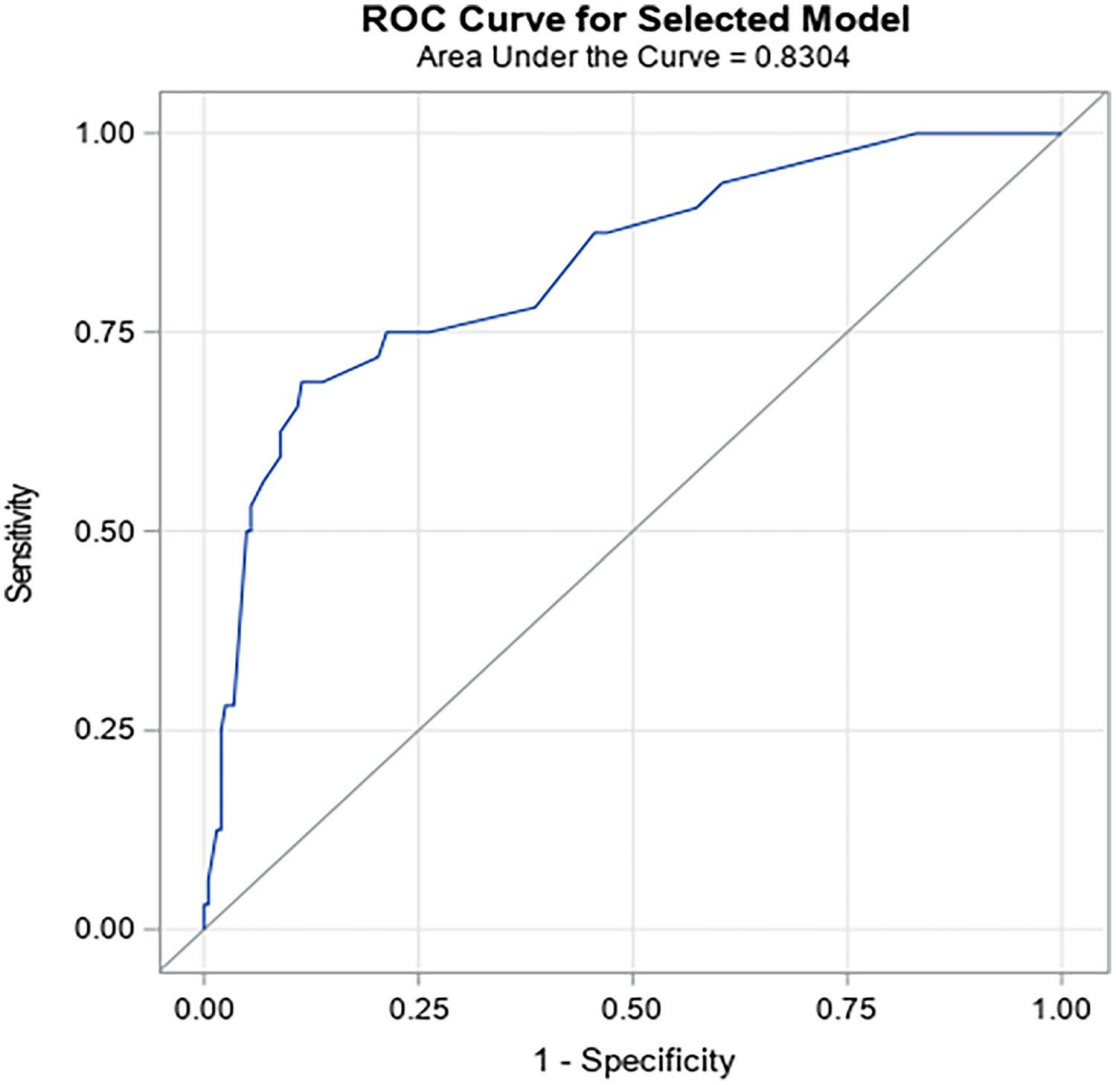
ROC curve related to the final multivariate model. ROC, receiver operating characteristic

Gestational age was a significant independent protecting factor for early CPAP failure (i.e., every one week increase, odds ratio (OR) = 0.703; 95% confidence interval (CI) 0.493–1.004; *p* = 0.0526). In contrast, a lower Apgar score at 10 min (i.e., every one-point decrease; OR = 1.725; 95% CI 1.148–2.591; *p* = 0.0086) was a significant independent risk factor for early CPAP failure (Table 2). Neither FiO_2_ nor FiO_2_*PEEP in the delivery room was significant risk factors for early CPAP failure, based on univariate analyses. However, FiO_2_*PEEP > 1.50 at 3 h (vs. ≤ 1.50; OR = 18.660; 95% CI 7.158–48.640; *p* < 0.0001) was a significant independent risk factor for early CPAP failure (Table 2). A higher FiO_2_*PEEP at 3 h was thus the strongest factor associated with CPAP failure. The overall model had an area under ROC curve of 0.83, suggesting that all variables combined together can predict early CPAP failure with increased accuracy (Table 2; Fig. 2).

## Discussion

To our knowledge, NEOBS is the first study to investigate the factors associated with CPAP failure, including maternal and neonatal clinical characteristics, type of pregnancy and delivery, respiratory parameters (maximum FiO_2_ and PEEP), and in moderate-to late preterm infants. In this subgroup analysis of the NEOBS study, 12% of moderate-to-late preterm infants had CPAP failure requiring mechanical ventilator support or surfactant administration. Of note, the rate of CPAP failure was lower in our study than in previous studies in extreme or very early preterm infants (20–45%) [12, 13]. This difference in CPAP failure rate could be due to differences in gestational age, patient selection criteria, and treatment approaches. The proportion of patients with FiO_2_ > 21% and FiO_2_*PEEP > 1.05, > 1.25, > 1.50, and > 1.80 at each time point during respiratory support was significantly higher among infants with CPAP failure versus those with CPAP success.

Our analysis identified the following key characteristics associated with CPAP failure among moderate-to-late preterm infants: type of pregnancy (singleton vs. multiple), decrease in Apgar score at 10 min, and FiO_2_ and FiO_2_*PEEP at 3 h of life. Using ROC curve analysis, we identified FiO_2_ with a low cut-off of 23% at 3 h after delivery as the strongest factor associated with CPAP failure. These findings in moderate-to-late preterm infants are consistent with those reported in earlier preterm infants (i.e., higher FiO_2_ at 2 h after delivery was a significant predictor of CPAP failure) [14]; notably, the FiO_2_ threshold in the previous study (29%) was higher than that in the current study (23%), perhaps reflecting the slightly different patient populations in terms of the degree of prematurity. In this study, the strongest factor associated with CPAP failure was the product of FiO_2_ and PEEP; the risk of CPAP increased 20 times in infants requiring FiO_2_*PEEP > 1.50 compared with those requiring FiO_2_*PEEP ≤ 1.50 at 3 h.

Previous studies have focused on the FiO_2_ threshold in preterm infants born at ≤ 32 WG and the association between FiO_2_ threshold and CPAP failure, including those by Dargaville and colleagues (FiO_2_ > 30% in the first few hours after birth in very preterm infants born at 25–32 WG) [13], De Jaegere and colleagues (FiO_2_ > 25% in the first couple of hours were significantly associated with CPAP failure in preterm infants born at < 30 WG) [15]; Rocha and colleagues (also in earlier preterm infants; FiO_2_ of 40% in the first 4 h of life was a significant predictor of CPAP failure in preterm infants born at 26–30 WG) [16], Murki and colleagues (FiO_2_ of 40% at CPAP initiation was a significant predictor of CPAP failure in infants born at ≤ 32 WG) [11], and Dell’Orto and colleagues (FiO_2_ of 23% was highly predictive of CPAP failure in infants born at 24–32 WG) [10]. Higher FiO_2_ requirements have also been shown to predict failure of bilevel positive airway pressure therapy in late preterm infants with respiratory distress [17]. Overall, the current analysis completes the findings of previous studies and extends knowledge to infants born moderate or late preterm.

Our analysis revealed no significant differences in maternal antenatal corticosteroid treatment between infants with CPAP failure and CPAP success. Although the antenatal use of corticosteroids, even at ≥ 34 WG, reduces neonatal respiratory morbidity [18], corticosteroids are generally not recommended in women at risk of preterm delivery beyond 34 WG [6].

Singletons appeared to be at a higher risk of early CPAP failure than twins in our univariate analysis, but this statistical association did not remain in the multivariate model. This may be due to differences in the underlying cause of preterm birth, as multiple pregnancy alone is a common cause of moderate preterm birth for twins; therefore, physicians were better prepared to manage the respiratory problems associated with premature birth. However, this is not the case for singletons, who might instead have a specific underlying risk factor (or factors, e.g., maternal factors such as smoking during pregnancy, maternal age, and hypertension or diabetes in pregnancy) [19], which could have additional impacts on respiratory health in the neonatal period. This is something that needs to be investigated further. Additionally, the risk of CPAP failure increased 1.7 times with every one-point decrease in Apgar score at 10 min; however, this factor may only be helpful for a few individual risk assessments, as the Apgar scores were > 7 at 10 min in the majority of infants.

In our analysis, we observed low rates of surfactant use in the delivery room. Early INSURE therapy has been shown to reduce the rate of CPAP failure in infants born at 33 to 36 + 6/7 WG [20]. In the same way, less invasive surfactant administration (LISA) has been shown to prevent early CPAP failure, even if most of the infants included were < 32 WG [21–23]. In this study, only four infants received surfactant via the LISA method, but they did not have CPAP failure. As previously reported, the centers including in the NEOBS study were only starting to use LISA method [9]. A new study incorporating the LISA method along with the recent guidelines [6, 7] would be interesting.

This study has several limitations. This is a post hoc analysis of data of 375 moderate-to-late preterm infants from an observational study; although associations can be determined, no conclusions can be drawn regarding causality. There is also limited external generalizability due to all study participants being recruited at level 2 and 3 maternity centers in a single high-income country (France). Nevertheless, there are limited data on respiratory failure in late preterm infants. The strength of this study is the large population size that included participants from most of the regions of France. Moreover, being an observational study, it reflects current clinical practice in France and its impact on clinical outcomes of infants. In addition, we used a respiratory score (FiO_2_*PEEP) rather than other calculations, such as the oxygenation index (mean airway pressure*FiO_2_*100/partial pressure of oxygen [PaO_2_]). Even if this score cannot assess severity of hypoxic respiratory failure, it is a good approximation of PaO_2_ and can be used in clinical practice to maintain a normal oxygen saturation for the newborn infant, according to international guidelines [6, 7]. Further epidemiological studies, machine learning algorithms, or new tools such as lung ultrasound are required for an optimal individual assessment of CPAP failure and early specific treatments, such as surfactants [24–26].

In conclusion, oxygen requirement during CPAP therapy, especially the product of FiO_2_ and PEEP, was an important factor associated with early CPAP failure in moderate-to-late preterm infants. The combination of singleton pregnancy, low Apgar score at 10 min, and high FiO_2_*PEEP at 3 h can predict early CPAP failure with increased accuracy. Our study also highlighted important areas for future research into the prediction or prevention of CPAP failure.

### Supplementary Information

Below is the link to the electronic supplementary material.

Supplementary file1 (DOCX 152 KB)

## Data Availability

Data generated/analyzed during the current study are available from the corresponding author upon reasonable request.

## Abbreviations

CI: Confidence interval
CNIL: Commission Nationale de l’Informatique et des Libertes
CPAP: Continuous positive airway pressure
CPP: Comité de Protection des Personnes
FiO_2_: Fraction of inspired oxygen
H_2_O: Dihydrogen oxide (water)
INSURE: Intubate surfactant extubate
LISA: Less invasive surfactant administration
OR: Odds ratio
pCO_2_: Carbon dioxide pressure
PEEP: Positive end-expiratory pressure
RDS: Respiratory distress syndrome
ROC: Receiver operating characteristics
USA: Unites States of America
WG: Weeks of gestation
