# Supplementary material for: Early factors associated with continuous positive airway pressure failure in moderate and late preterm infants
Source: Eur J Pediatr. 2023 Sep 26;182(12):5399–407. doi: 10.1007/s00431-023-05090-1 (PMC10746609; doi:10.1007/s00431-023-05090-1)

**Early factors associated with continuous positive airway pressure failure in moderate and late preterm infants**

***European Journal of Pediatrics***

**Online Resource 1**

**Authors:** Pierre Tourneux, Thierry Debillon, Cyril Flamant, Pierre-Henri Jarreau, Benjamin Serraz, Isabelle Guellec

**Corresponding author:** Prof. Pierre Tourneux
Neonatal Intensive Care Unit, University Hospital of Amiens, 80054 Amiens cedex 1, France.
**Email:** [Tourneux.Pierre@chu-amiens.fr](mailto:Tourneux.Pierre@chu-amiens.fr)

**Supplementary Table S1** Fraction of inspired oxygen (FiO_2_) categories

|  | **CPAP success (n=330)** | | **CPAP failure (n=45)** | | **p-value** | |
| --- | --- | --- | --- | --- | --- | --- |
| **FiO_2_max in delivery room, n (%)** |  |  | |  | |  |
| >22% | 184 (63) | 32 (78) | | 0.08 | |  |
| >23% | 183 (62.7) | 32 (78) | | 0.06 | |  |
| >24% | 183 (62.7) | 32 (78) | | 0.06 | |  |
| >25% | 170 (58.2) | 28 (68.3) | | 0.24 | |  |
| >26% | 170 (58.2) | 28 (68.3) | | 0.24 | |  |
| >27% | 170 (58.2) | 27 (65.9) | | 0.4 | |  |
| >28% | 170 (58.2) | 27 (65.9) | | 0.4 | |  |
| >29% | 170 (58.2) | 27 (65.9) | | 0.4 | |  |
| >30% | 99 (33.9) | 17 (41.5) | | 0.38 | |  |
| >35% | 96 (32.9) | 13 (31.7) | | 1 | |  |
| >40% | 62 (21.2) | 12 (29.3) | | 0.24 | |  |
| **FiO_2_max at 3 hours, n (%)** |  |  | |  | |  |
| >22% | 82 (25.6) | 28 (66.7) | | <0.0001 | |  |
| >23% | 67 (20.9) | 28 (66.7) | | <0.0001 | |  |
| >24% | 64 (20) | 27 (64.3) | | <0.0001 | |  |
| >25% | 45 (14.1) | 24 (57.1) | | <0.0001 | |  |
| >26% | 44 (13.8) | 21 (50) | | <0.0001 | |  |
| >27% | 42 (13.1) | 20 (47.6) | | <0.0001 | |  |
| >28% | 36 (11.3) | 20 (47.6) | | <0.0001 | |  |
| >29% | 35 (10.9) | 20 (47.6) | | <0.0001 | |  |
| >30% | 13 (4.1) | 18 (42.9) | | <0.0001 | |  |
| >35% | 4 (1.3) | 16 (38.1) | | <0.0001 | |  |
| >40% | 1 (0.3) | 12 (28.6) | | <0.0001 | |  |

*CPAP* continuous positive airway pressure, *FiO_2_max* maximum FiO_2_.

**Supplementary Table S2** Detailed ventilatory support data at each time point

| **Total patients (32–36 WG)** | **CPAP success (n=330)** | | | | | **CPAP failure (n=45)** | | | | | **p-value** | | | | |
| --- | --- | --- | --- | --- | --- | --- | --- | --- | --- | --- | --- | --- | --- | --- | --- |
|  | **DR** | **3h** | **6h** | **12h** | **24h** | **DR** | **3h** | **6h** | **12h** | **24h** | **DR** | **3h** | **6h** | **12h** | **24h** |
| No ventilatory support, n (%) | 0 | 41 (12.4) | 57 (17.3) | 83 (25.2) | 124 (37.6) | 0 | 2 (4.4) | 1 (2.2) | 2 (4.4) | 7 (15.6) |  |  |  |  |  |
| **Ventilatory support** |  |  |  |  |  |  |  |  |  |  |  |  |  |  |  |
| Max FiO_2_, mean±SD (%) | 35.8±20.6 | 22.8±4.1 | 22.2± 3.3 | 21.9± 2.8 | 22±3.5 | 42.9±27.1 | 34.4±15.9 | 30.1±14 | 25±6.5 | 25.2±7.4 | 0.10 | <0.0001 | <0.0001 | <0.0001 | <0.001 |
| Max FiO_2_ <30%, n (%) | 122 (41.8) | 285 (89.1) | 308 (95.4) | 313 (96) | 303 (94.4) | 14 (34.1) | 22 (52.4) | 26 (61.9) | 34 (77.3) | 35 (77.8) | 0.4 | <0.0001 | <0.0001^b^ | <0.0001^b^ | <0.01^b^ |
| Max FiO_2_ ≥30%, n (%) | 170 (58.2) | 35 (10.9) | 15 (4.6) | 13 (4) | 18 (5.6) | 27 (65.9) | 20 (47.6) | 16 (38.1) | 10 (22.7) | 10 (22.2) |  |  |  |  |  |
| FiO_2_max >21%, n (%) | 184 (63) | 84 (26.3) | 64 (19.8) | 50 (15.3) | 47 (14.6) | 32 (78) | 28 (66.7) | 25 (59.5) | 17 (38.6) | 15 (33.3) | 0.08 | <0.0001 | <0.0001 | <0.001 | <0.01 |
| PEEP, mean±SD (cmH_2_O) | 5.1±1.2 | 4.9±0.8 | 4.9±0.8 | 4.9±1.4 | 4.9±1.6 | 5±0.5 | 5.2±0.9 | 4.8±0.8 | 4.9±0.7 | 5±0.8 | 0.97 | 0.08 | 0.58 | 0.64 | 0.19 |
| PEEP <6 cmH_2_O, n (%) | 196 (87.1) | 194 (85.5) | 189 (85.9) | 162 (83.9) | 125 (82.8) | 22 (88) | 23 (69.7) | 28 (82.4) | 29 (80.6) | 27 (79.4) | 0.9 | 0.02 | 0.59^b^ | 0.63^b^ | 0.64 |
| PEEP ≥6 cmH_2_O, n (%) | 29 (12.9) | 33 (14.5) | 31 (14.1) | 31 (16.1) | 26 (17.2) | 3 (12) | 10 (30.3) | 6 (17.6) | 7 (19.4) | 7 (20.6) |  |  |  |  |  |
| Max FiO_2_*PEEP | n=217 | n=227 | n=220 | n=193 | n=151 | n=25 | n=33 | n=34 | n=36 | n=34 |  |  |  |  |  |
| Max FiO_2_*PEEP, mean±SD | 1.9±1.1 | 1.1±0.3 | 1.1± 0.3 | 1.1±0.4 | 1.1±0.4 | 2.5±1.4 | 1.8±0.9 | 1.4± 0.6 | 1.2± 0.5 | 1.3± 0.6 | <0.01 | <0.0001 | 0.01 | 0.21 | 0.09 |
| Max FiO_2_*PEEP >1.05 (0.21x5), n (%) | 156 (71.9) | 78 (34.4) | 67 (30.5) | 58 (30.1) | 53 (35.1) | 24 (96) | 22 (66.7) | 16 (47.1) | 12 (33.3) | 15 (44.1) | <0.01 | <0.001 | 0.08 | 0.7 | 0.33 |
| Max FiO_2_*PEEP >1.25 (0.25x5 or 0.21x6), n (%) | 139 (64.1) | 52 (22.9) | 48 (21.8) | 45 (23.3) | 44 (29.1) | 19 (76) | 20 (60.6) | 15 (44.1) | 10 (27.8) | 12 (35.3) | 0.27 | <0.0001 | <0.01 | 0.53 | 0.54 |
| Max FiO_2_*PEEP >1.5 (0.30x5 or 0.25x6), n (%) | 84 (38.7) | 17 (7.5) | 13 (5.9) | 9 (4.7) | 13 (8.6) | 16 (64) | 19 (57.6) | 10 (29.4) | 5 (13.9) | 6 (17.6) | 0.02 | <0.0001 | <0.001 | 0.05 | 0.13 |
| Max FiO_2_*PEEP >1.8 (0.30x6), n (%) | 73 (33.6) | 8 (3.5) | 5 (2.3) | 4 (2.1) | 5 (3.3) | 12 (48) | 12 (36.4) | 6 (17.6) | 5 (13.9) | 5 (14.7) | 0.19 | <0.0001 | <0.01 | <0.01 | 0.02 |
| Aggressive CPAP support^a^, n (%) |  |  |  |  |  |  |  |  |  |  |  |  |  |  |  |
| Yes |  | 37 (12.1) | 37 (11.7) | 32 (10.3) | 26 (8.2) |  | 10 (25) | 7 (17.1) | 8 (18.6) | 7 (16.7) |  | 0.02 | 0.33 | 0.11 | 0.07 |
| No |  | 270 (87.9) | 279 (88.3) | 278 (89.7) | 293 (91.8) |  | 30 (75) | 34 (82.9) | 35 (81.4) | 35 (83.3) |  |  |  |  |  |

*CPAP* continuous positive airway pressure, *DR* delivery room, *FiO_2_* inspired oxygen fraction, *max* maximum, *PEEP* maximum positive end-expiratory pressure, *SD* standard deviation, *WG* weeks of gestation.

^a^Aggressive CPAP support was defined as intermittent positive pressure ventilation or positive expiratory pressure ≥6 cmH_2_O, and no aggressive CPAP support was defined as positive expiratory pressure <6 cmH_2_O or high-flow nasal cannula oxygen therapy or no support.
^b^Fisher’s test.

**Supplementary Table S3** Treatment approaches in infants with early continuous positive airway pressure therapy success and failure (univariate analysis)

|  | **CPAP success (n=330)** | **CPAP failure (n=45)** | **Total analyzed population**  **(n=375)** | **p-value** |
| --- | --- | --- | --- | --- |
| **Surfactant administration in DR, n (%)** | 8 (2.4) | 4 (8.9) | 12 (3.2) | 0.04 |
| Surfactant technique used |  |  |  |  |
| LISA method | 4 (50.0) | 0 | 4 (33.3) | - |
| INSURE method | 1 (12.5) | 1 (25.0) | 2 (16.7) | - |
| Intubation tube with MV | 0 | 2 (50.0) | 2 (16.7) | - |
| Missing data | 3 (37.5) | 1 (25.0) | 4 (33.3) | - |
| **Initial destination, n (%)** |  |  |  |  |
| Initial admission at site of birth | 306 (92.7) | 35 (77.8) |  | <0.01 |
| Direct transfer to another center | 24 (7.3) | 10 (22.2) |  |  |
| **Initial admission at birth site, n (%)** |  |  |  |  |
| Maternity unit | 6 (1.8) | 0 |  |  |
| Resuscitation unit | 136 (41.2) | 20 (44.4) |  |  |
| Intensive care unit | 121 (36.7) | 6 (13.3) |  |  |
| Neonatal unit | 42 (12.7) | 9 (20.0) |  |  |
| Missing data | 25 (7.6) | 10 (22.0) |  |  |
| **Surfactant administration before 24 hours, n (%)** | 21 (6.4) | 35 (77.8) | 56 (14.9) | <0.0001 |
| Surfactant technique used |  |  |  |  |
| LISA method | 10 (47.6) | 3 (8.6) | 13 (23.2) |  |
| INSURE method | 8 (38.1) | 2 (5.7) | 10 (17.9) |  |
| Intubation tube with MV | 0 | 30 (85.7) | 30 (53.6) |  |
| Missing data | 3 (14.3) | 1 (2.9) | 4 (7.1) |  |
| **Caffeine administration before 24 hours, n (%)** | 103 (31.2) | 18 (40.0) | 121 (32.3) | 0.24 |
| **Umbilical venous catheter, n (%)** | 69 (22.5) | 22 (51.2) | 91 (26.1) | <0.0001 |

*CPAP* continuous positive airway pressure, *DR* delivery room, *INSURE* INtubation SURfactant Extubation, *MV* mechanical ventilation, *LISA* less invasive surfactant administration.

**Supplementary Fig. 1** Receiver operating characteristic (ROC) curve and cut-off values for (a) maximum fraction of inspired oxygen (FiO_2_max), (b) FiO_2_max at each cut-off, (c) fraction of inspired oxygen (FiO_2_)*positive end-expiratory pressure (PEEP), and (d) FiO_2_*PEEP at each cut-off at 3 hours (H3).
*AUC* area under the curve.


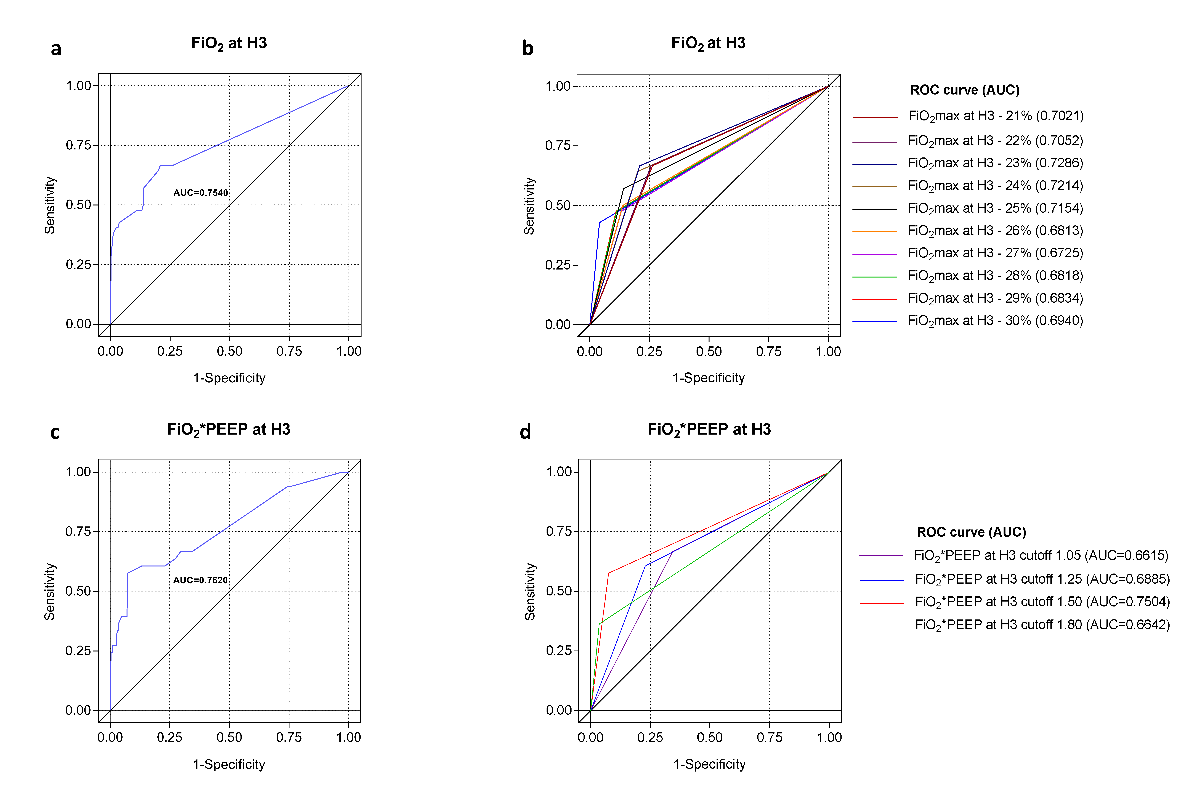

Supplement: Supplementary file 1 — Supplementary file1 (DOCX 152 KB) [file 431_2023_5090_MOESM1_ESM.docx]
